# Acute Music‐Frequency Exposure Modulates Salivary Stress and Neurotrophic Markers in Young Adults: A Randomized Controlled Trial

**DOI:** 10.1002/brb3.71452

**Published:** 2026-04-27

**Authors:** Ümmü Gülşen Bozok, Gülbahar Böyük Özcan, Bülent Bayraktar, Doğukan Özen

**Affiliations:** ^1^ Faculty of Medicine, Department of Physiology Ankara Medipol University Ankara Türkiye; ^2^ Faculty of Health Sciences, Department of Physiotherapy and Rehabilitation Bayburt University Bayburt Türkiye; ^3^ Faculty of Veterinary Medicine, Department of Biostatistics Ankara University Ankara Türkiye

**Keywords:** BDNF, cognitive function, CREB, GRP78, music, stress, Stroop test

## Abstract

**Background:**

The exam period is a period full of intense stress and anxiety that deeply affects cognitive and physiological responses for university students. Listening to music has the potential to protect cellular health and alleviate the negative effects of stress by affecting individuals' mood, and cognitive functions. This study aimed to investigate the differential effects of 528 and 432 Hz music frequencies on salivary BDNF, CREB, and GRP78 levels in young adults, along with their association with cognitive performance under exam‐related stress.

**Methods:**

A total of 162 healthy university students were randomly assigned to three groups: no music (control), 528 Hz instrumental music, and 432 Hz instrumental music. Participants listened to music for 20 min, after which saliva samples were collected and analyzed for brain‐derived neurotrophic factor (BDNF), glucose‐regulated protein 78 (GRP78) and cyclic AMP response element‐binding protein (CREB) levels using ELISA. Cognitive performance was assessed using the Stroop word‐color test administered by medical doctor.

**Results:**

Exposure to 528 Hz music significantly increased salivary BDNF (3.84 [2.76–4.13]) and CREB levels (1.5 [1.38–1.59]) compared to control (*p* < 0.05), whereas GRP78 levels were elevated in the 432 Hz group (0.51 [0.47–0.58]) (*p* < 0.05). No significant differences were observed between groups in Stroop test performance.

**Conclusion:**

These findings suggest that specific music frequencies elicit distinct biological responses under exam‐related stress, even in the absence of immediate cognitive performance differences. Salivary neurotrophic and stress‐related biomarkers may offer valuable non‐invasive insights into music's neuromodulatory effects.

**Trial Registration:**

ClinicalTrials.gov identifier: AMU‐EC‐8522

## 1 Introduction

Music plays an important role in various relaxation techniques to make exam stress more manageable and support cognitive performance (Twitchell et al. 2025). It exerts its effects primarily via the hippocampus, mesolimbic reward system, and cortical networks. The hippocampus regulates emotional processing of music, while phase synchronization supports music recognition and working memory (Trost and Frühholz 2015; Ara and Marco‐Pallarés 2020). Music perception in the brain correlates with activity in the ventral striatum and nucleus accumbens (Salimpoor et al. 2013). Nevertheless, the specific effects of music on cognitive performance vary based on individual differences and music genre (Dunn et al. 2012). Although music is frequently used in stress management, few studies have compared specific frequencies in terms of biological correlates under real‐life stressors such as exams. Listening to music before a stressor can modify the psychobiological stress response, primarily by facilitating autonomic recovery (Eskine et al. 2020). Neuroimaging reviews further document music‐evoked modulation of limbic and hypothalamic structures implicated in affect and stress regulation (Koelsch 2014), and the neurochemistry pathways through which music engages systems for reward, arousal/stress, immunity and affiliation (Chanda and Levitin 2013). But, direct mechanistic evidence for specific tunings remains limited. Small human studies contrasting 432 versus 440 Hz report modest differences in autonomic indices such as heart rate/blood pressure (Calamassi and Pomponi 2019). A recent double‐blind randomized pilot in healthcare workers suggests improvements in anxiety and some vital signs with 432 Hz, underscoring the need for controlled trials (Calamassi et al. 2022). A total of 528 Hz is the best frequency because it stops the formation of reactive oxygen radicals in studies (Babayi Daylari et al. 2019); additionally, 432 Hz is favored due to its capacity to promote health benefits (Calamassi and Pomponi 2019). Prior studies suggest that music tuned to 528 Hz may reduce oxidative stress, while 432 Hz has been associated with autonomic relaxation and physiological benefits (Calamassi and Pomponi 2019; Babayi and Riazi 2017). However, music influences a range of cognitive processes beyond memory, including attention, problem‐solving, learning, reasoning, and creativity (Eskine et al. 2020). Engaging with music before commencing work enhances creative cognition.

BDNF affects synaptic plasticity and neuronal survival and is used to assess cognitive performance (Dinoff et al. 2016; Szuhany et al. 2015). BDNF is highly expressed in the cerebral cortex and hippocampus (Mohammed et al. 2002). In particular, physical exercise has been associated with increased hippocampal volume, improved memory and executive function, and reduced risk of cognitive impairment, all of which are supported by neuroplasticity facilitated by BDNF (Dinoff et al. 2016; Szuhany et al. 2015). In addition to BDNF, CREB also play a key role in learning and memory. Neuronal CREB is found particularly in hippocampus and cortex (Tanis et al. 2008). It contributes to learning and memory processes, enhancing cognitive capabilities through the modulation of BDNF expression (Tanis et al. 2008; Kida and Serita 2014). In addition to neuroplasticity markers, controlling cellular stress is also important for cognitive function. GRP78 is a sign of stress in the endoplasmic reticulum (ER) and is very important for keeping proteins in balance (Brocchieri et al. 2008). Moreover, GRP78 decreases in neurodegenerative diseases (White et al. 2007). In addition to its role as a stress biomarker, GRP78 also plays a protective role in cellular homeostasis by regulating ER stress responses and promoting protein folding mechanisms (Lee 2005).

Students may be preoccupied during exam periods and struggle to focus. There is limited solid scientific evidence evaluating the effects of these on neurobiological stress markers and cognitive outcomes. Using simple methods like music provides both convenience and comfort for students. Furthermore, the idea of achieving this process in a very short time is appealing to students. Therefore, this study aimed to evaluate the acute effects of listening to 528 and 432 Hz music on neuroplasticity and salivary biomarkers of stress (BDNF, CREB, and GRP78) in university students. Furthermore, by assessing the impact of these frequencies on cognitive performance during exam periods, we provided a small scientific stepping stone, especially for those short on time and searching for an easy method.

## 2 Materials and Methods

### 2.1 Participants

This research was performed with volunteer university students aged 18–25 enrolled at Ankara Medipol University, Faculty of Health Sciences. This cross‐sectional and relational type research was conducted in the Central Anatolia Region of Türkiye in October 2024. After determining the number of students with proportional stratification in sample selection, simple random sampling was applied. Participation in the study was entirely voluntary, and informed consent was acquired from all individuals.

**FIGURE 1:**
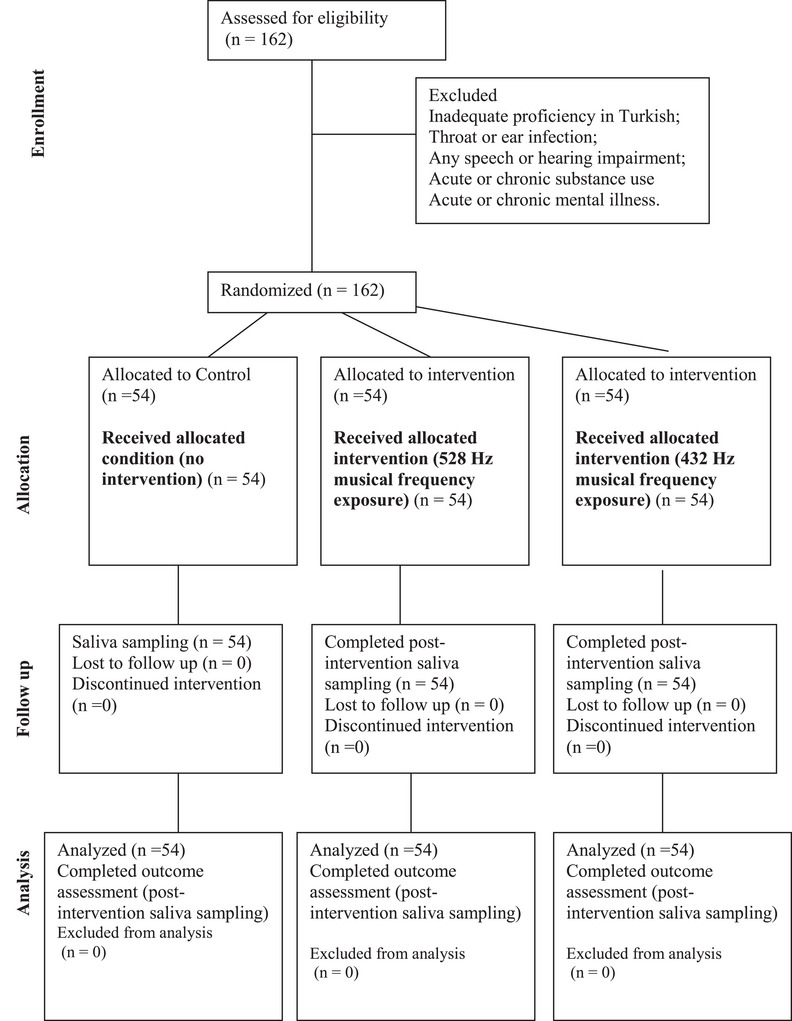
CONSORT flow diagram of participant progress through the randomized controlled trial. After screening 162 individuals, participants were randomized in equal numbers (*n* = 54 per arm) to control (no intervention), 528‐Hz musical frequency exposure, or 432‐Hz musical frequency exposure. All randomized participants received the allocated condition and completed post‐intervention saliva sampling, and none were lost to follow‐up or discontinued the intervention; therefore, all participants were included in the final analysis. All procedures were conducted under identical environmental conditions without external cues.

The sample size was computed with G*Power software (Faul et al. 2007) to estimate the minimum required sample size, using an effect size (*f*) = 0.25 (Cohen 2013), power (1‐beta) = 0.8, and type 1 error rate level (alpha) = 0.05 for three groups. The calculations indicated that a minimum of 159 participants is required for the experiment. In this study, a total of 162 university students participated (Figure 1). The study participants were randomly allocated into three distinct groups. Participants were assigned in a 1:1:1 ratio to Control (no intervention), 432 Hz, or 528 Hz using a manual permuted‐block randomization. Group 1 (Control): Music was not played during the experiment. Group 2 (528 Hz Group): Participants listened to instrumental music at a frequency of 528 Hz for 20 min through headphones. Group 3 (432 Hz Group): Participants listened to instrumental music at a frequency of 432 Hz for 20 min through headphones (Gan et al. 2016). There were no significant differences between groups in age or sex distribution and in pre‐intervention outcomes (salivary markers) (all *p* > 0.05), indicating homogeneous groups prior to the intervention. All groups were evaluated under identical environmental settings and were not influenced by external cues throughout the experimental intervention. The inclusion criteria for the study were (1) Turkish as the native language, (2) enrollment as a university student aged 18–25, (3) possessing the requisite cognitive ability for communication, and (4) no clinically relevant hearing impairment. The primary rationale for concentrating on this age demographic is that university students exhibit a preference for music, and cognitive functions, particularly memory, are critical during this stage of development (Eskine et al. 2020). Exclusion criteria were (1) lack of proficiency in Turkish; (2) presence of a throat or ear infection 1 week prior to and/or during the study period; (3) acute and/or chronic substance usage; (4) acute or chronic mental illness (Figure 1).

### 2.2 Analysis of Cognitive Function

**FIGURE 2:**
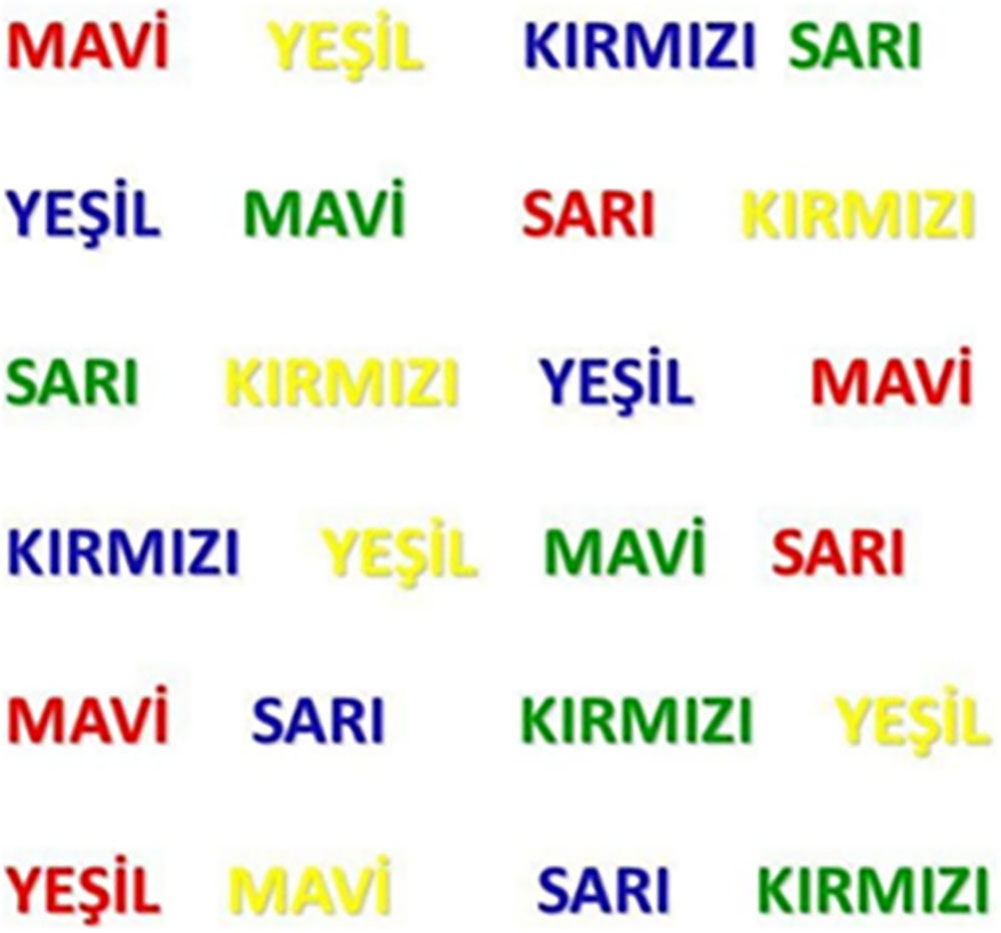
Stroop color‐word test. In this test, participants are presented with color names printed in incongruent ink colors (e.g., the word “BLUE” written in red ink). They are initially instructed to read the word aloud while their response time is recorded. In the next stage, participants are asked to identify the ink color instead of reading the word, and the duration is measured. This task assesses cognitive strain by comparing color identification time to the automatic reading of words. Our study specifically employed Turkish characters. In this version, participants encountered color names in Turkish and were required to either read the words (e.g., MAVİ [BLUE], KIRMIZI [RED]) or identify the ink color. This adaptation allows for a more targeted cognitive processing and selective attention assessment.

The Stroop Color‐Word Test is a prevalent experimental assessment in cognitive psychology, utilized to evaluate selective attention and inhibitory control (Nasiri et al. 2023). Group 1 served as the control group without music, Group 2 was subjected to 20 min of 528 Hz instrumental music, and Group 3 experienced 20 min of 432 Hz instrumental music (Gan et al. 2016). Following music exposure, the Stroop color‐word test was conducted for each group to evaluate cognitive function (Henik 1996). It has been widely used to assess cognitive impairments in psychiatric and neurological conditions (Pilli et al. 2013), to investigate the neurocognitive basis of selective attention (Rebai et al. 1997), and to examine age‐related declines in inhibitory control (West and Alain 2000). Given that computer technologies offer superior accuracy in measuring and controlling stimulus presentation compared to traditional pen‐and‐paper assessments, this study presents a straightforward and dependable method for evaluating the central nervous system effects of music (Figure 2). Initially, participants were instructed to read the words, and their reading times were documented. Then, participants were instructed to identify the colors of various incongruent color‐word pairs, and reading time was documented. Participants were prohibited from progressing to the subsequent word unless the color of each word was accurately articulated (Pilli et al. 2013). The assessment of cognitive function was conducted by comparing word reading and color reading times (Yuan et al. 2020). The Stroop test was administered individually by a medical doctor and lasted approximately 10 min.

### 2.3 Collection and Analysis of Saliva Samples

The passive drooling technique is considered the gold standard when collecting saliva samples for biological testing because it allows researchers to preserve saliva samples for further analysis (Baek et al. 2025). The presence of BDNF (Kırbaş et al. 2025), CREB (Okur 2025) and GRP78 (Kirbaş et al. 2024) in saliva samples offers great potential for research and clinical applications as it is a non‐invasive sampling method.

Saliva and data collection took place on the university's official exam dates. Sessions were scheduled ∼60 min prior to each participant's exam and conducted in rolling 8:00 a.m.–11:00 a.m. morning blocks on those dates. After the 20‐min exposure (528 Hz, 432 Hz, or no‐music control), unstimulated saliva (passive drool) was collected immediately after the music intervention and before the Stroop test, under identical environmental conditions, prior to the exam.

Unstimulated saliva was collected by passive drool into Sarstedt (Germany) tubes. Samples were centrifuged at 1500 × *g* for 15 min (NF‐1200R, Nüve, Turkey), filtered, and stored at −80°C until analysis. Salivary BDNF, GRP78, and CREB were quantified by sandwich sandwich enzyme‐linked immunosorbent assay (ELISA) according to the manufacturers’ instructions. For BDNF, we used the Human BDNF ELISA Kit (BT LAB, E1302Hu, China) with a calibration range of 31.25–2000 pg/mL; intra‐assay and inter‐assay coefficients of variation were 8% and 10%, respectively. For GRP78, we used the Human GRP78 ELISA Kit (Sun Long, SL2048Hu, China) with a limit of detection of 16 pg/mL. For CREB, we used the Human CREB ELISA (BT LAB, SG‐11988, China) with a 31.25–2000 pg/mL working range and 8% and 10% intra‐/inter‐assay coefficients of variation. All samples were run in duplicate; absorbance was read at 450 nm, and concentrations were calculated from the standard curve.

### 2.4 Statistical Analysis

Descriptive statistics were calculated and reported as either Median (first Quartile [Q1]‐3rd Quartile [Q3])″, depending on the distribution of the data. Prior to conducting hypothesis tests, the data were subjected to a Shapiro–Wilk test to determine whether the data conformed to a normal distribution and a Levene's test to assess the homogeneity of variances as part of the parametric test assumptions. The statistical significance of the differences between the control, 432 Hz, and 528 Hz groups was determined by Kruskal–Wallis analysis, which was conducted to assess the results of the Stroop test and salivary parameters. In cases where a significant difference was identified, the Dunn–Bonferroni test was employed as a post‐hoc test. The *p* < 0.05 criterion was utilized in all statistical comparisons. Data analysis was conducted using the SPSS 30 package program (Meng et al. 2018) (IBM Corp. Released 2024).

## 3 Results

### 3.1 Examination of Cognitive Variables: Neurotrophic Factors and Stress Markers

BDNF, CREB, and GRP78 levels among the control group and the groups exposed to 432 and 528 Hz music are presented in Table 1. All neurotrophic parameters differed significantly between groups (*p* < 0.05). Distinct letters (a, b, and c) within the same column denote statistically significant disparities. Significant group differences in BDNF levels were noted between the groups (*p* < 0.05). Upon analysis of the median values, it was noted that the control group (3.54 [2.58–3.78]) and the 528 Hz group (3.84 [2.76–4.13]) exhibited comparable values (a); however, the 432 Hz group (2.88 [2.01–3.59]) was significantly lower than the groups mentioned above (b).

**TABLE 1:** Comparison of Mean Salivary BDNF, CREB, and GRP78 (ng/mL) values of participants in the study groups (mean ± SD).

	Group	Arithmatic mean ± SD (s)	Median (Q1–Q3) (s)	p**
**BDNF**	**Control**	3.29 ± 0.93	3.54 (2.58–3.78)a	0.013*
	**432 hz**	2.9 ± 0.99	2.88 (2.01–3.59)b	
	**528 hz**	3.44 ± 1.01	3.84 (2.76–4.13)a	
**CREB**	**Control**	1.14 ± 0.23	1.13 (1.02–1.26)b	<0.001*
	**432 hz**	1.04 ± 0.28	1.06 (0.88–1.19)c	
	**528 hz**	1.48 ± 0.16	1.5 (1.38–1.59)a	
**GRP78**	**Control**	0.46 ± 0.07	0.45 (0.42–0.51)b	<0.001*
	**432 hz**	0.51 ± 0.08	0.51 (0.47–0.58)a	
	**528 hz**	0.43 ± 0.06	0.44 (0.39–0.47)c	

*Note*: Different letters in the same column indicate statistically significant differences (*p* < 0.05). Data are presented as mean ± standard deviation (SD) and median (Q1–Q3).

Group comparisons of salivary BDNF, CREB, and GRP78 concentrations following exposure to different music frequencies. Exposure to 528 Hz music was associated with increased BDNF and CREB levels, while 432 Hz music elevated GRP78 levels, suggesting divergent neuromodulatory and stress‐related responses.

Abbreviations: BDNF, brain‐derived neurotrophic factor; CREB, cAMP response element‐binding protein; GRP78, glucose‐regulated protein 78.

**Kruskal–Wallis, *p < 0.05 statistically significant, a,b,c:

Significant differences in CREB levels were observed between the groups (*p* < 0.001). The CREB levels in the 528 Hz group (1.5 [1.38–1.59]) were significantly elevated compared to the other two groups, the control group (1.13 [1.02–1.26]) and the 432 Hz group (1.06 [0.88–1.19]).

Significant changes were seen across the groups regarding GRP78 levels (*p* < 0.001). GRP78 levels in the 432 Hz group were significantly higher (0.51 [0.47–0.58]) than the control group (0.45 [0.42–0.51]) and the 528 Hz group (0.44 [0.39–0.47]) (Table 1).

### 3.2 Cognitive Performance Outcomes: Stroop Examination

The data in Table 2 compares Stroop test outcomes among the control group, 432 Hz group, and 528 Hz group during the word and color reading phases. The mean ± SD for the word reading phase were 8.13 ± 1.68, 8.23 ± 2.00, and 8.17 ± 1.77 s for the control, 432 Hz, and 528 Hz groups, respectively. During the color reading phase, the mean and standard deviation for the control group were 13.78 ± 2.51, for the 432 Hz group 15.01 ± 3.39, and for the 528 Hz group 14.54 ± 2.96 s, respectively. The impact of music at varying frequencies on Stroop test performance, revealing no statistically significant differences between the groups in any test phase (*p* > 0.05). Although mean values in the 432 and 528 Hz groups were numerically higher than those in the control group during the color reading phase, these differences were not statistically significant (Table 2).

**TABLE 2:** Comparison of Stroop test results of participants in study groups (mean ± SD).

	Group	Arithmatic mean ± SD (s)	Median (Q1–Q3) (s)	p**
**Text reading**	**Control**	8.13 ± 1.68	7.78 (7.06–9.13)	0.970
	**432 hz**	8.23 ± 2	7.77 (6.98–8.98)	
	**528 hz**	8.17 ± 1.77	7.86 (6.71–9.43)	
**Color reading**	**Control**	13.78 ± 2.51	13.62 (11.68–15.34)	0.156
	**432 hz**	15.01 ± 3.39	14.48 (12.59–16.66)	
	**528 hz**	14.54 ± 2.96	14.25 (12.14–15.75)	

*Note*: There was no significant difference between the groups listening to 432 and 528 Hz music in terms of Stroop word‐color test (*p* > 0.05). Data are presented as mean ± standard deviation (SD) and median (Q1–Q3). Comparison of Stroop test performance across study groups exposed to different music frequencies. No statistically significant differences were observed between the groups in either text reading or color naming components of the Stroop task (*p* > 0.05). These findings suggest that brief exposure to 432 Hz or 528 Hz music does not acutely affect cognitive interference performance in healthy individuals.

**Kruskal–Wallis, *p < 0.05 statistically significant, Q1–Q3: first to third quartile.

## 4 Discussion

Music affects cognitive processes that regulate, structure, and manage many cognitive functions, such as attention, working memory, organizing, problem‐solving, and mental flexibility, including task switching (Chan et al. 1998). Neuroimaging research corroborates these behavioral findings, demonstrating heightened brain activation in musicians relative to non‐musicians (Pallesen et al. 2010). Music‐based applications can positively influence anxiety and depression symptoms, enhancing cognitive skills including verbal fluency, working memory, and recognition (Moschonas et al. 2023).

Correlation studies between saliva and blood and/or urine indicate that saliva is a dependable diagnostic specimen that may be readily collected for steroids, some hormones, various medications, and antibodies (Hofman 2001). BDNF is essential for neuronal development, survival, and synaptic plasticity. Reduced levels of BDNF correlate with cognitive deterioration, depression, and neurodegenerative disorders, whereas elevated levels are deemed crucial for learning, memory, and neuronal function. (Colucci‐D'Amato et al. 2020; Azman and Zakaria 2022). Our study found that 432 Hz music reduced BDNF levels, potentially adversely affecting cognitive functions. Conversely, the 528 Hz music frequency was found to sustain or elevate BDNF levels, indicating a potential neuroprotective effect (Table 1). Research on musicians has demonstrated a correlation between plasma BDNF levels and cerebral BDNF levels (Klein et al. 2011). Our findings demonstrated that elevated BDNF levels in saliva correlate with music exposure. This may offer mechanistic insight into how auditory stimuli influence neurotrophic regulation, even in non‐musicians. Furthermore, the use of salivary BDNF as a measurable endpoint provides a non‐invasive neurobiochemical rationale for assessing brain plasticity (Schlaug 2001).

CREB is a protein required for synaptic plasticity and learning and memory functions (Barco et al. 2003). This protein enhances synaptic connections by elevating the expression of neurotrophic factors, including BDNF, in neurons (Amidfar et al. 2020). High CREB activity promotes neural plasticity and long‐term memory, whereas low CREB levels are associated with cognitive impairment and progression of neurodegenerative diseases (Li et al. 2024). Our investigation indicates that the notable elevation in CREB levels triggered by 528 Hz music implies that this frequency may enhance synaptic plasticity and learning capacity (Table 1). Conversely, 432 Hz music was found to diminish CREB levels to their minimum (Table 1). Our study showed that 528 Hz increased both BDNF and CREB levels, which are known to be associated with cognitive performance. These results may indicate that the 432 Hz frequency could have unfavorable effects on cognitive performance. A reduction in CREB levels may adversely affect long‐term memory formation, learning capacity, and neural plasticity. Reduced CREB levels, notably linked to neurodegenerative disorders (El‐Shimi et al. 2024), indicate that the impact of the 432 Hz frequency on cognitive functions warrants additional examination. While no research explicitly connects music to CREB activation, Chaudhury et al. (2009) revealed that auditory stimulation during the embryonic phase fosters hippocampal growth and amplifies CREB activation (Chaudhury and Wadhwa 2009).

The Stroop test was developed by John Ridley Stroop in 1935 and is a common and valuable neuropsychological test for assessing cognitive performance (Smith 2009) (Figure 2). Cognitive performance was evaluated by comparing the differences between plain text and colored text reading and analyzing the color discrimination process from plain text with the Stroop test. Nonetheless, no substantial difference was observed between short‐term listening to 528 and 432 Hz music and cognitive function as measured by attention (Table 2). For this reason, it is thought that short‐term listening is not sufficient to create measurable changes in the brain or cognitive performance. (Yuan et al. 2020) assessed task performance by analyzing feedback on coffee response time and accuracy rate using behavioral experiments involving 31 healthy participants using the Stroop test. The study's findings indicate that coffee consumption may positively influence cognitive processes. Cognitive flexibility, attentional control, and executive functioning, as assessed by the Stroop test, were observed to enhance following coffee consumption (Yuan et al. 2020). Although salivary BDNF, CREB, and GRP78 levels significantly changed following music frequency exposure, these biochemical alterations were not reflected in the immediate Stroop test outcomes. This may be due to the temporal nature of neuroplasticity: while BDNF and CREB are central to memory and synaptic modulation, their effects on cognitive function often require sustained or repeated stimulation to result in behavioral changes (Liao et al. 2025). Hence, the Stroop test may not capture such early‐phase molecular shifts in acute settings.

GRP78, also known as binding immunoglobulin protein or HSPA5, is a critical chaperone protein found in the ER of cells. In the event of intracellular stress, especially ER stress, the level of GRP78 increases significantly (Espina et al. 2023). Our data indicates that the notable elevation in GRP78 levels caused by 432 Hz music may activate the cellular stress response or exacerbate ER stress (Table 1). The increase in GRP78 levels may indicate the cells' adaptive response to environmental stressors. The discovery that elevated salivary GRP78 levels prior to the Stroop test may produce results comparable to a cortisol response supports this assertion (Iqbal et al. 2023). Significantly, increased GRP78 levels have been linked to neurodegenerative diseases over time, underscoring the necessity for a thorough examination of the potential impacts of the 432 Hz frequency on cellular stress mechanisms. The markedly reduced GRP78 levels in the 528 Hz music group, relative to both the control and 432 Hz groups (Table 1), indicate that this frequency may inhibit the cellular stress response and promote ER homeostasis. The decrease in GRP78 levels may suggest that cells are undergoing diminished stress and that biological systems are operating more harmoniously. This indicates that music with a frequency of 528 Hz may diminish ER stress and so alleviate cellular stress, ultimately providing supportive and neuroprotective effects. The GRP78 elevation observed in the 432 Hz group may not solely reflect increased cellular stress, but could also be interpreted as an adaptive mechanism, suggesting that the 432 Hz music may have initiated a protective unfolded protein response aiming to preserve cellular homeostasis. GRP78 plays a protective role by facilitating proper protein folding, preventing protein aggregation, and attenuating apoptotic signals (Lee 2005). This dual role warrants further mechanistic investigation. Therefore, the elevated GRP78 levels observed in the 432 Hz group may not solely reflect detrimental stress responses but could also indicate an attempt by the cell to restore proteostasis through an adaptive unfolded protein response. This interpretation aligns with findings that GRP78 upregulation is often associated with resilience against stress insults (Akinyemi et al. 2023).

This study examines the impact of music on cognitive function, utilizing 528 and 432 Hz frequencies recognized. Especially, acoustic features—particularly pitch height and related spectral properties—shift arousal, which in turn can modulate autonomic and endocrine responses relevant to salivary markers. Experimental work shows pitch height systematically alters perceived arousal/tension (Ilie and Thompson 2006). Prior research, including investigations by K. Akimoto et al., has shown that music tuned to 528 Hz markedly alleviates tension within a few minutes of exposure (Akimoto et al. 2018). In parallel, our investigation also revealed that 528 Hz music markedly decreased GRP78 levels in comparison to the control group (Table 1). Thoma et al., who comprehensively examined the effects of music on the endocrine, autonomic, cognitive, and affective domains of the human stress response, evaluated salivary cortisol and salivary alpha‐amylase, heart rate, respiratory sinus arrhythmia, perceived stress, and anxiety. Using parameters complementary to ours, they showed that listening to music before a standardized stressor primarily modulates the autonomic nervous system, and to a lesser extent the endocrine and psychological components of the stress response (Thoma et al. 2013). In this context, our study provides an additional step toward understanding the beneficial effects of music on the human body.

Our study also has certain limitations. First, the sample group consisted solely of university students aged 18–25, which limits the generalizability of the findings to broader age groups and individuals with different demographic characteristics. Additionally, the music exposure was restricted to 20 min, and long‐term effects were not assessed. The potential influence of participants' individual music preferences and prior musical experiences on cognitive responses was not controlled. Given that music perception is highly subjective, the impact of individual differences on neurobiological responses should be examined in greater detail. Furthermore, since biochemical analyses were conducted using salivary samples, they may not fully reflect biochemical changes in the central nervous system. Therefore, future research should integrate more comprehensive biochemical analyses using cerebrospinal fluid or blood samples, along with neuroimaging techniques, to provide a more detailed understanding. This study's primary strength is in its controlled and randomized experimental methodology, which examines the impact of music frequencies on neurotrophic indicators. This study offers a comprehensive view of the neurobiological impacts of music by integrating biochemical and cognitive tests to analyze the influence of music exposure on cognitive functions.

## 5 Conclusion

Our current study has revealed the effects of 432 and 528 Hz music frequencies on cerebral (BDNF and CREB) and stress response (GRP78) in university students during the exam period. Our study is a short‐term study, a “pilot” study to generate prospective hypotheses and identify methodological issues in a relatively new or under‐researched area of the effects of music frequencies on cerebral biomarkers and stress response. Overall, the 528 Hz condition was consistent with a more neurotrophic profile, with higher CREB, BDNF comparable to control, and lower GRP78, whereas the 432 Hz condition was associated with a less neurotrophic profile, with lower BDNF and CREB and higher GRP78 stress effect. Stroop test performance did not differ significantly across frequency groups, and thus this cognitive measure did not corroborate the frequency related differences observed in salivary biomarkers. So, comprehensive studies, in which relevant biomarkers are examined at the molecular level and with long‐term experimental periods, are needed.

## Author Contributions

Ü.G.B and B.B. designed the study. Ü.G.B. and G.B.Ö. collected data. B.B. did the sample measurement. D.Ö. analyzed the data. Ü.G.B.and B.B. prepared the draft plan. All authors contributed to writing the manuscript. All authors read and approved the final manuscript.

## Ethics Statement

The research was approved by the Ankara Medipol University Non‐Interventional Ethics Committee (7.10.2024/ Decision no: 129). Before the data were collected, the volunteers were informed about the study in accordance with the Declaration of Helsinki and their written/verbal consent was obtained.

## Funding

The authors have nothing to report.

## Conflicts of Interest

The authors decalre no conflicts of interest.

## Data Availability

The corresponding author upon reasonable request will provide data supporting the findings of this study.
